# Contribution of Disulfide Bridges to the Thermostability of a Type A Feruloyl Esterase from *Aspergillus usamii*

**DOI:** 10.1371/journal.pone.0126864

**Published:** 2015-05-13

**Authors:** Xin Yin, Die Hu, Jian-Fang Li, Yao He, Tian-Di Zhu, Min-Chen Wu

**Affiliations:** 1 Key Laboratory of Carbohydrate Chemistry and Biotechnology, Ministry of Education, School of Biotechnology, Jiangnan University, Wuxi, China; 2 State Key Laboratory of Food Science and Technology, School of Food Science and Technology, Jiangnan University, Wuxi, China; 3 Wuxi Medical School, Jiangnan University, Wuxi, China; University Paris South, FRANCE

## Abstract

The contribution of disulfide bridges to the thermostability of a type A feruloyl esterase (AuFaeA) from *Aspergillus usamii* E001 was studied by introducing an extra disulfide bridge or eliminating a native one from the enzyme. MODIP and DbD, two computational tools that can predict the possible disulfide bridges in proteins for thermostability improvement, and molecular dynamics (MD) simulations were used to design the extra disulfide bridge. One residue pair A126-N152 was chosen, and the respective amino acid residues were mutated to cysteine. The wild-type AuFaeA and its variants were expressed in *Pichia pastoris* GS115. The temperature optimum of the recombinant (re-) AuFaeA^A126C-N152C^ was increased by 6°C compared to that of re-AuFaeA. The thermal inactivation half-lives of re-AuFaeA^A126C-N152C^ at 55 and 60°C were 188 and 40 min, which were 12.5- and 10-folds longer than those of re-AuFaeA. The catalytic efficiency (*k*_cat_/*K*_m_) of re-AuFaeA^A126C-N152C^ was similar to that of re-AuFaeA. Additionally, after elimination of each native disulfide bridge in AuFaeA, a great decrease in expression level and at least 10°C decrease in thermal stability of recombinant AuEaeA variants were also observed.

## Introduction

A disulfide bridge is formed by the oxidation of two thiols each from two cysteines, thus linking the two cysteines and their respective main peptide chains, which can restrict the motion of the unfolded, random coil of protein or stabilize the folded state of protein [1,2]. One disulfide bridge can contribute 2.3–5.2 kcal/mol to the thermodynamic stability of proteins [1,3]. The contribution of disulfide bridges to the stability of proteins can be measured, to a certain extent, by the change in protein thermostability upon introduction or elimination of one or more disulfide bridges. Considerable evidence has demonstrated the thermostability effects of engineered disulfide bridges in protein. For example, upon introduction of a disulfide bridge A162C-K308C in the lipase B (CalB) from *Candida antarctica*, the half-life of the enzyme was increased by 4.5-fold at 50°C [4]. The optimum temperature of xylanase (TLX) from *Thermomyces lanuginosus* increased approximately 10°C upon introduction of a disulfide bridge Q1C-Q24C [5]. Conversely, the absence of a disulfide bridge contributed to the increased conformational flexibility and thermolability of the lipase (PFL) from *Pseudomonas fragi* [6,7].

Feruloyl esterases (FAEs, EC 3.1.1.73) cleave the ester bond between polysaccharides and hydroxycinnamic acid in hemicellulose networks for the subsequent hydrolysis of hemicellulose by hemicellulose-digesting enzymes. Their potential for degradation and reutilization of the natural biomass is significant [8]. Based on substrate preference and primary structure homology, FAEs have been classified into four types: type A, B, C and D [9]. Hitherto, crystallographic structure of a type A FAE from *Aspergillus niger* has been analyzed. The structure of this enzyme is based on an α/β hydrolase fold and consists of a major nine-stranded mixed β-sheet, two minor two-stranded β-sheet arrangements and seven helixes [10,11]. Moreover, there are three disulfide bridges located in the FAE simulating three legs of a tripod [10]. FAEs that are similar to other biomass-degradation enzymes can be applied in several industries, such as animal feed preparation, papermaking, baking, biofuel, and production of bioactive phenolic components [12,13]. Unfortunately, most wild-type FAEs have poor thermostability, which lowers their tolerance to the high temperatures encountered in bioprocesses, as for example in pulp bleaching and feedstuff preparation. Only a limited number of thermostable FAEs have been reported thus far, and these FAEs are mostly from bacteria, such as type A TtFAE from *Thermoanaerobacter tengcongensis*, and type B Tx-Est1 from *Thermobacillus xylanilyticus* [14,15]. Generally, the FAEs from fungi are not thermostable, such as the *Af*FaeA from *Aspergillus flavus* and AnFaeA from *A*. *niger* [16,17]. Accordingly, it is very important to discover more thermophilic FAEs or to improve the thermostability of mesophilic enzymes and proteins by employing the promising strategy of protein engineering [18,19].

The thermostability of AnFaeA was improved through multiple amino acid substitutions by error prone PCR technique [20]. Some rational design methods have been developed and applied to increase the protein thermostability. The lipase CalB was thermally improved by rational design based on the flexibility of the amino acid residues (B-factor values) and RosettaDesign [21]. The rational engineering of disulfide bridges in protein is another promising strategy that has been used to improve the thermostability of T4 lysozyme [22], and *Trichoderma reesei* endo-1,4-β-xylanase II [23]. These rational protein engineering methods could greatly decrease the heavy workloads of the researcher. In our previous work, a gene *AufaeA* encoding a mesophilic type A FAE (AuFaeA) from *A*. *usamii* was cloned and expressed in *Pichia pastoris* [24]. In the present study, the contribution of disulfide bridges to AuFaeA thermostability was studied by introducing an extra disulfide bridge designed by computational prediction or eliminating a native one from the enzyme. This work made a first step for further studies on higher thermostability modification of type A FAEs, especially those from fungi, by other methods, such as N- or C-terminus substitution [25,26] and directed evolution [27].

## Materials and Methods

### Strains, Plasmids and Culture Media

*Escherichia coli* JM109 and plasmid pUCm-T (Sangon, Shanghai, China) were used for gene cloning and DNA sequencing. A recombinant T-plasmid, pUCm-T-*AufaeA*, was constructed and preserved in our laboratory [24]. *E*. *coli* DH5α and plasmid pPIC9K (Invitrogen, San Diego, CA, USA) were used for constructing the recombinant expression plasmids. *E*. *coli* JM109 and DH5α were cultured in the LB medium (10 g/L tryptone, 5 g/L yeast extract and 10 g/L NaCl, pH 7.2). *P*. *pastoris* GS115 and its transformants were cultured and methanol-induced in the YPD, MD, YPD with geneticin G418, BMGY and BMMY media, which were prepared as described in the manual of Multi-Copy Pichia Expression Kit (Invitrogen).

### Analysis of Primary and Three Dimensional Structures

Homology sequence search at the NCBI website (http://www.ncbi.nlm.nih.gov/) was performed using the BLAST server, while homology alignment of type A FAE primary structures was analyzed using the ClustalW2 program (http://www.ebi.ac.uk/Tools/msa/clustalw2/). Using the crystal structure of type A FAE from *A*. *niger* (AnFaeA, PDB code: 1USW) as a template, three dimensional (3D) structures of *A*. *usamii* AuFaeA and its variants, sharing high primary-structure identities with *A*. *niger* AnFaeA, were homologically modeled and optimized using the MODELLER 9.9 program (http://salilab.org/modeller/). The B-factor values of AnFaeA (1USW) were analyzed by B-FITTER software [28]. The 3D structure was visualized using PyMOL software (http://pymol.org).

### Location of the Disulfide Bridge Sites

Modeling of Disulfide Bonds in Proteins (MODIP, http://caps.ncbs.res.in/dsdbase/modip.html) and Disulfide by Design (DbD, http://cptweb.cpt.wayne.edu/DbD2/) were used to detect amino acid pairs, in the 3D structure of AuFaeA, where the disulfide bridges were likely to form. Based on the physicochemical properties of the located amino acids and their positions in the 3D structure of AuFaeA, several pairs of amino acids were selected for substitution with cysteine residues, creating a series of candidate variants of AuFaeA.

### Introduction of an Extra Disulfide Bridge

The root mean square deviation (RMSD) value is an important index for estimating the thermostability of a protein conformation. RMSD was defined as the C_α_-atomic fluctuation parameter of a protein from its original conformation to the changed one at a high temperature and at a certain time. In addition, RMSD had a negative correlation with the thermostability of a protein [29]. To predict the thermostability of AuFaeA and its candidate variants with extra disulfide bridges, their 3D structures were modeled and subjected to molecular dynamics (MD) simulation processes, respectively, at 500 K for 10 ns using GROMACS 4.5 package (http://www.gromacs.org/). The RMSD value was calculated using g_rms software from GROMACS 4.5 package, and statistical analysis was performed using Origin 9 software (http://www.originlab.com/).

Based on the computational prediction, AuFaeA^A126C-N152C^, a variant of AuFaeA, with the smallest RMSD value was selected. The variant-encoding gene, *AufaeA* ^A126C-N152C^, was constructed by synchronously mutating Ala^126^- and Asn^152^-encoding codons of *AufaeA* into Cys^126^- and Cys^152^-encoding ones. Using pUCm-T-*AufaeA* as a template, *AufaeA* ^A126C-N152C^ was amplified using a QuikChange Mutagenesis Kit (Stratagene, La Jolla, CA, USA) with two pairs of forward-reverse PCR primers, A126C and N152C (Table 1), and inserted into plasmid pUCm-T. The resultant recombinant T-plasmid, named pUCm-T-*AufaeA* ^A126C-N152C^, was transformed into *E*. *coli* JM109 and confirmed by DNA sequencing.

**Table 1:** Primers used for site-directed mutagenesis.

Primer	Primer sequence (5’-3’)^a^	Size (bp)
A126C	ATCCGGACTATTGCCTTACCGTGACA	26
	TGTCACGGTAAGGCAATAGTCCGGAT	26
N152C	GCGACATATGACTGCGTCCGTCTGTAC	27
	GTACAGACGGACGCAGTCATATGTCGC	27
C29T	ACGCCGACCTAACTAATATTCCATCGACT	29
	AGTCGATGGAATATTAGTTAGGTCGGCGT	29
C91T	ACTCTACCTCAAACTAACGATTGCG	25
	CGCAATCGTTAGTTTGAGGTAGAGT	25
C234T	ACTGGGGATGAAGTACAGACTTGTGAGGCA	30
	TGCCTCACAAGTCTGTACTTCATCCCCAGT	30

^a^ The mutant codons are boxed.

### Elimination of the Native Disulfide Bridges

The contribution of native disulfide bridges to the thermostability of AuFaeA was investigated by constructing three single variants: AuFaeA^C29T^, AuFaeA^C91T^ and AuFaeA^C234T^. These mutations were designed by substituting three cysteine residues, Cys^29^, Cys^91^ and Cys^234^, with the corresponding threonine ones, respectively. Their encoding genes, *AufaeA* ^C29T^, *AufaeA* ^C91T^ and *AufaeA* ^C234T^, were constructed by site-directed mutagenesis with three pairs of forward-reverse primers C29T, C91T and C234T (Table 1), respectively, using a QuikChange Mutagenesis Kit. The target PCR products were separately purified, and then inserted into pUCm-T. The resultant recombinant T-plasmids, named pUCm-T-*AufaeA* ^C29T^,-*AufaeA* ^C91T^ and-*AufaeA* ^C234T^, were transformed into *E*. *coli* JM109 and confirmed by DNA sequencing.

### Enzyme Activity and Protein Assays

The substrate, *p*-nitrophenyl ferulate (*p*NPF), was synthesized with a single-step method as reported previously [30]. FAE activity was determined by measuring the amount of *p*-nitrophenol (*p*NP) released from *p*NPF as described [31], with minor modification. Briefly, the reaction mixture (8 volumes of 100 mM Na_2_HPO_4_–citric acid buffer (pH 5.5) containing 2.5% (v/v) Triton X-100, 1 volume of 10 mM *p*NPF in dimethyl sulfoxide and 1 volume of suitably diluted enzyme) was incubated at 40°C for 10 min. The released *p*NP was measured at 410 nm using a spectrophotometer. One unit (U) of FAE activity was defined as the amount of enzyme that released 1 μmol *p*NP per minute under the standard assay conditions as stated above.

Sodium dodecyl sulfate-polyacrylamide gel electrophoresis (SDS-PAGE) was performed using the method by Laemmli [32]. The separated peptide bands were visualized by staining with Coomassie Brilliant Blue R-250 (Sigma, St. Louis, MO, USA), and molecular weights were estimated in comparison to the standard protein markers using Quantity One software. The protein content was measured with a BCA-200 Protein Assay Kit (Pierce, Rockford, IL, USA), using bovine serum albumin as the standard.

### Expression and Purification of the re-FAEs

*AufaeA* and its mutant genes were excised from their recombinant T-plasmids by digestion with *Eco*RI and *Not*I, respectively. The isolated genes were inserted into pPIC9K, and then transformed into *E*. *coli* DH5α. The resultant recombinant expression plasmids, designated as pPIC9K-*AufaeA*,-*AufaeA* ^A126C-N152C^,-*AufaeA* ^C29T^,-*AufaeA* ^C91T^ and-*AufaeA* ^C234T^, were separately linearized with *Sal*I and electroporated into *P*. *pastoris* GS115. All *P*. *pastoris* transformants were respectively inoculated on YPD plates with increasing concentrations of G418 for the screening of multiple copies of integrated FAE genes. *P*. *pastoris* transformed with pPIC9K without *AufaeA* or any of the mutants was used as a control (*P*. *pastoris* GSC). Expression of respective FAE genes in *P*. *pastoris* was performed according to the instructions of Multi-Copy Pichia Expression Kit (Invitrogen) with minor modification [33].

The transformed *P*. *pastoris* was induced by 1% methanol for 72 h, and the expressed re-FAE in the supernatant was salted out by adding ammonium sulfate (NH_4_)_2_SO_4_ to 75% saturation. The collected precipitate was dissolved and dialyzed in 20 mM Na_2_HPO_4_–citric acid buffer (pH 5.5). The dialyzed solution was concentrated by ultrafiltration using a 10-kDa cut-off membrane (Millipore, Billerica, MA, USA) and was loaded on a Sephadex G-50 column (Amersham Pharmacia Biotech, Uppsala, Sweden; 1.6 × 80 cm), followed by elution with the same buffer at a flow rate of 0.4 mL/min. Aliquots of 2 mL eluent containing re-FAE were pooled and concentrated for further studies.

### Thiol Titration

Guanidine hydrochloride (5.5 M, Sigma) was added for 15 min to denature the purified re-AuFaeA or re-AuFaeA^A126C-N152C^ (final concentration of 2 mg/mL) [34]. Thiol titration was performed by incubating thirty parts of denatured re-FAE with one part of 4 mg/mL dithionitrobenzoic acid in 0.25 M Tris-HCl buffer (pH 8.0) for 15 min at 25°C, and the absorbance was measured at 410 nm in a spectrophotometer [35]. The wild-type re-AuFaeA containing one free cysteine at position 235 was used as a control. The free cysteine content of each re-FAE was calculated from a cysteine concentration-OD_410_ standard formula, which was established by testing the OD_410_ of cysteine concentration at a range from 0 to 0.4 mM. The number of disulfide bridges in the re-FAEs was deduced from the different value of the number of total cysteines in protein primary structure and free ones in 3D structure at the tested concentration.

### Temperature Optimum and Thermal Inactivation Half-life

Purified re-AuFaeA and its variants were functioned in the standard enzyme activity assay conditions except the changed temperatures to measure the temperature optima, and were incubated in the absence of substrate at different temperatures for 60 min or longer time to estimate their thermostability. The thermal inactivation half-life (*t*_1/2_) was defined as the time when the residual activity of the re-FAE, determined under the standard conditions, was 50% of its original activity.

### Enzyme Kinetic Parameters

The hydrolytic reaction rates (U/mg) of re-AuFaeA and re-AuFaeA^A126C-N152C^ were separately determined under the standard assay conditions, with a wide-range of *p*NPF concentration (0.5 to 20.0 mM). Data were fitted to the Michaelis-Menten equation to generate *K*_m_ and *k*_cat_ values, using Graph-Pad Prism 5.0 software.

## Results and Discussion

### Location of the Disulfide Bridge Sites

**Fig 1:**
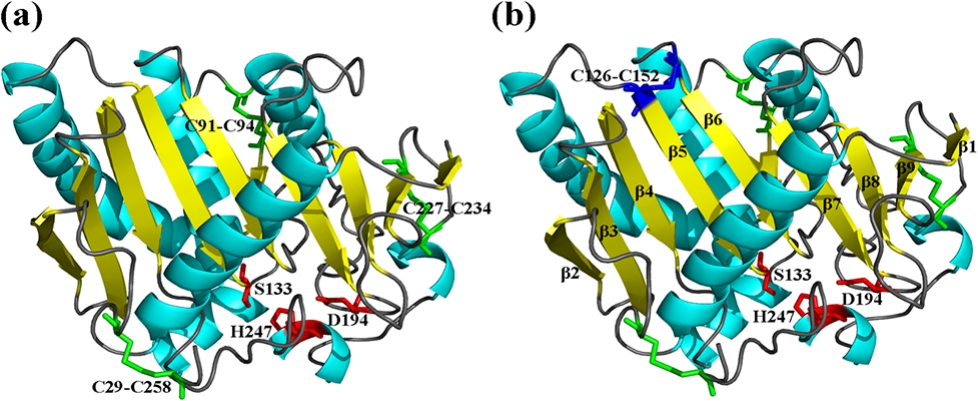
The modeled 3D structures of AuFaeA and AuFaeA^A126C-N152C^. The 3D structures of AuFaeA and AuFaeA^**A126C-N152C**^ were modeled using the crystal structure of AnFaeA (PDB code: 1USW) as template. (a) The catalytic triad S133-H247-D194 and three native disulfide bridges C29-C258, C91-C94, C227-C234 are separately marked in red and green. (b) The extra disulfide bridge C126-C152 located in AuFaeA^**A126C-N152C**^ is marked in blue.

**Fig 2:**
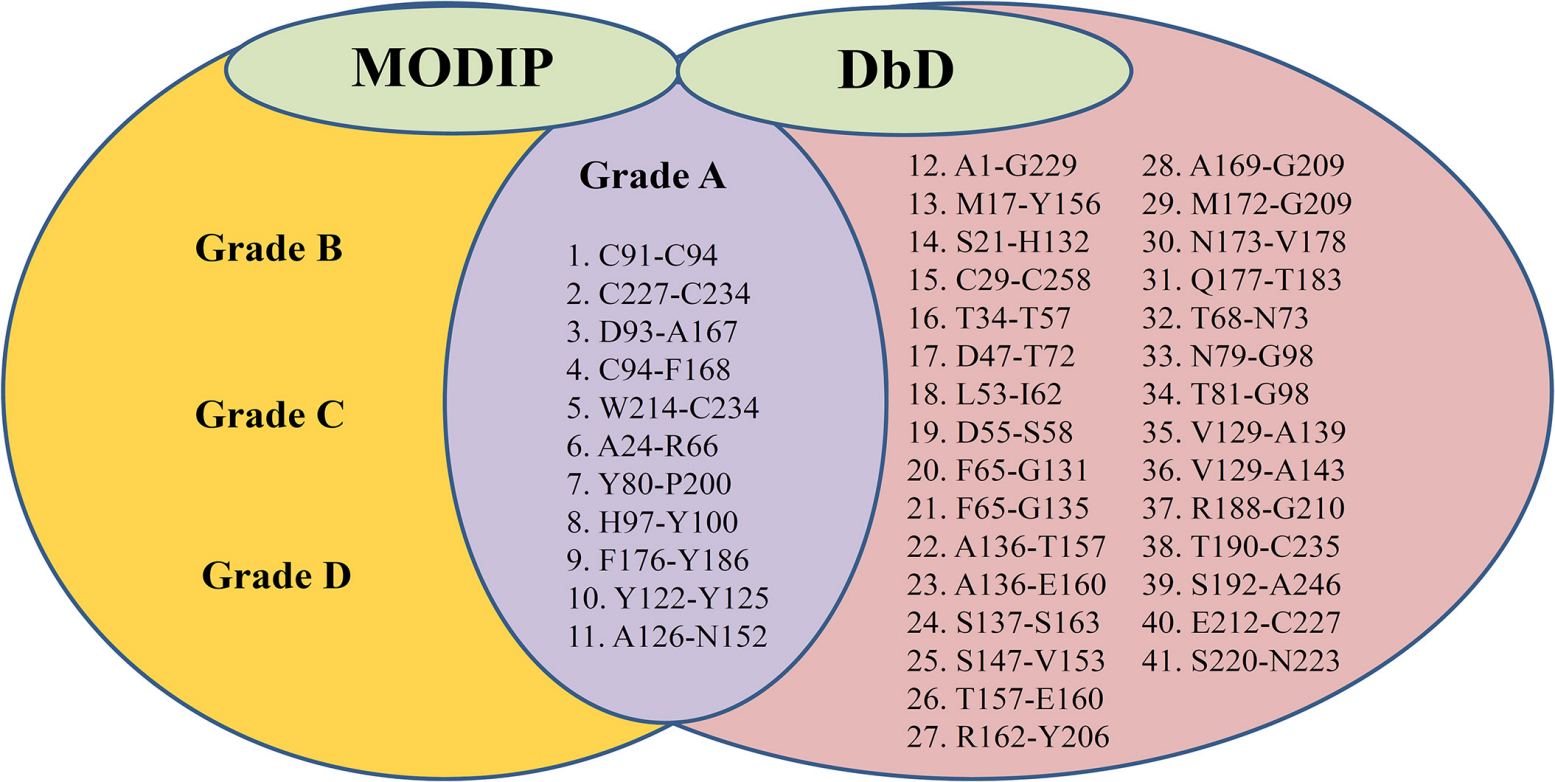
Alignment of amino acid sites for disulfide bridges predicted by MODIP and DbD. The amino acid sites predicted by DbD are shown in the right (pink) oval. The amino acid sites predicted by MODIP are shown in the left (yellow) oval. The middle (purple) oval shows 11 amino acid sites predicted by both DbD and MODIP that have the highest possibilities of disulfide bridge formation.

The primary structures of the AuFaeA and AnFaeA (PDB code: 1USW) were highly homologous (98.5%) in identity. Identification of three native disulfide bridges, C29-C258, C91-C94 and C227-C234, in AuFaeA (Fig 1A), was an essential step for engineering the disulfide bridges to improve AuFaeA thermostability. DbD and MODIP have been previously applied to successfully improve the stability of xylanase from *Bacillus stearothermophilus*, lipase from *Rhizomucor miehei* and acetylcholinesterase from *Drosophila melanogaster* [35–37]. In this study, DbD suggested 41 pairs of amino acid sites for possible disulfide bridges (Fig 2), while 69 amino acid pairs were ranked from grades A to D by MODIP (data not shown) based on their likelihood to form disulfide bridges. Eleven pairs of amino acids were identified by both DbD and MODIP for their highest possibilities to form disulfide bridges (Fig 2). Upon further analysis, 6 predicted pairs, C91-C94, C227-C234, D93-A167, C94-F168, W214-C234 and H97-Y100, were rejected as follows: the first two pairs were native disulfide bridges; the following three pairs were located near the native ones and could affect the protein conformation; and the last pair was located near a catalytic triad with a distance of 6 Å, which could influence the enzymatic activity. Ultimately, the remaining 5 pairs, A24-R66, Y80-P200, F176-Y186, Y122-Y125 and A126-N152, were evaluated by MD simulation.

### Introduction of the Extra Disulfide Bridge

**Fig 3:**
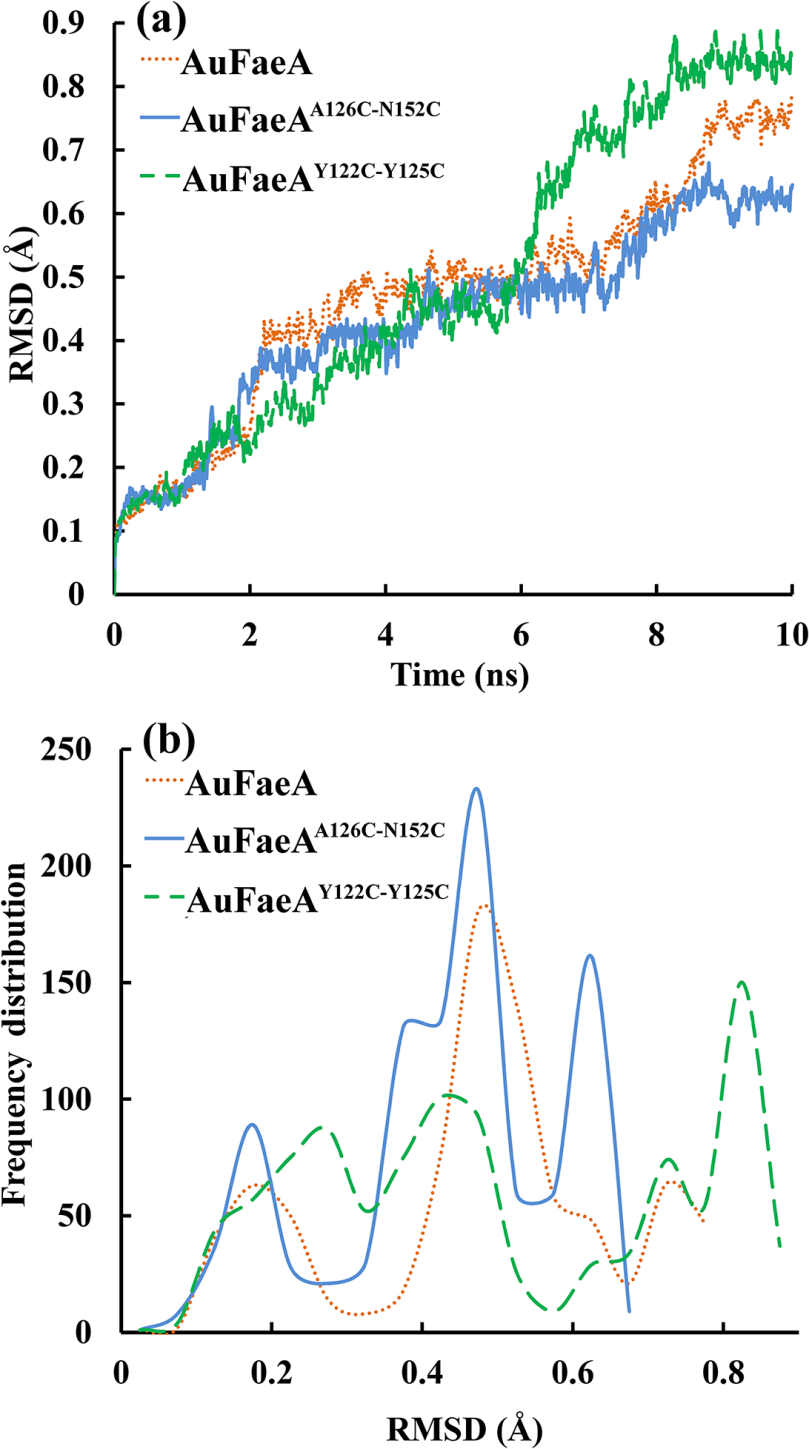
Calculation and distribution of the RMSD values. Graphical representation of: (a) RMSD values of AuFaeA (dotted line), AuFaeA^**A126C-N152C**^ (solid line), and AuFaeA^**Y122C-Y125C**^ (dashed line), respectively, after MD simulation processes at 500 K for 10 ns. (b) Distribution of RMSD values of AuFaeA (dotted line), AuFaeA^**A126C-N152C**^ (solid line), and AuFaeA^**Y122C-Y125C**^ (dashed line). MD, molecular dynamics; RMSD, root mean square deviation.

The RMSD value is an important index for evaluating the conformational flexibility of protein at high temperature [29]. A hybrid xylanase AEXynM, with an increased melting temperature (*T*_m_) of 34°C, was more thermostable than its wild type, which was consistent with the evaluation of RMSD values [26]. In this study, the RMSD values of AuFaeA^A24C-R66C^, AuFaeA^Y80C-P200C^, AuFaeA^F176C-Y186C^ and AuFaeA^Y122C-Y125C^ (selected as an example shown in Fig 3) were higher, while those of AuFaeA^A126C-N152C^ were lower than those of AuFaeA, after equilibration (Fig 3A). Simultaneously, the distributions of RMSD values of AuFaeA^A126C-N152C^, AuFaeA^Y122C-Y125C^ and AuFaeA were statistically analyzed and were mainly concentrated on 0.475 Å, 0.825 Å and 0.525 Å, respectively (Fig 3B). The data indicated that AuFaeA^A126C-N152C^ was less flexible and more rigid than AuFaeA and AuFaeA^Y122C-Y125C^. Because the rigidity of a protein was positively related to its thermostability [38], the variant AuFaeA^A126C-N152C^ was predicted to be more thermostable than the wild-type AuFaeA. Therefore, the amino acid pair A126-N152 was selected for substitution with cysteine residues to construct the extra disulfide bridge in AuFaeA. After site-directed mutagenesis, the mutant gene was obtained and confirmed by DNA sequencing.

### Expression and Purification of the re-AuFaeA and re-AuFaeA^A126C-N152C^

**Fig 4:**
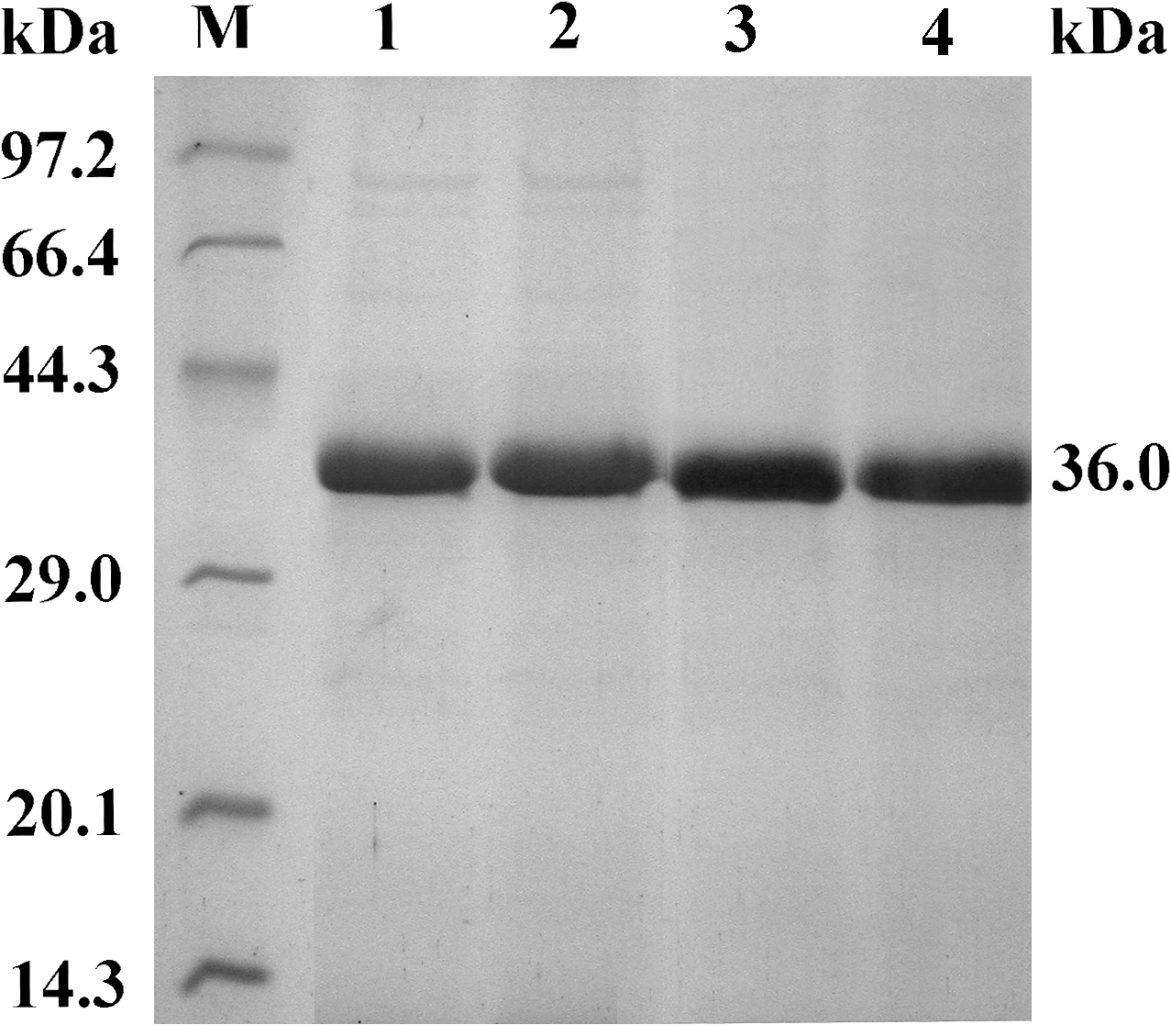
SDS-PAGE analysis of the re-AuFaeA and re-AuFaeA^A126C-N152C^. Lane M, standard protein marker; lane 1 and 2, the cultured supernatants of re-AuFaeA and re-AuFaeA^**A126C-N152C**^; lane 3 and 4, the purified re-AuFaeA and re-AuFaeA^**A126C-N152C**^ with same molecular weights of 36.0 kDa.

An advantage of the *P*. *pastoris* expression system is the high purity of the expressed recombinant protein, as described in the Multi-Copy Pichia Expression Kit (Invitrogen, USA). Purities of recombinant *A*. *usamii* xylanase and *A*. *sulphureus* β-mannanase expressed in *P*. *pastoris* GS115 and X-33 have been reported to be as high as 90 and 97%, respectively [39,40]. In this work, after the transformed *P*. *pastoris* was induced by 1.0% methanol for 72 h, the content of expressed re-AuFaeA or re-AuFaeA^A126C-N152C^ was more than 88% in the cultured supernatant (Fig 4, lane 1 and 2). The activities of the expressed re-AuFaeA and re-AuFaeA^A126C-N152C^ were 11.03 and 13.73 U/mL, respectively, which were slightly lower than that (16.6 U/mL) of re-AnFaeA [41]. The specific activities of purified re-AuFaeA and re-AuFaeA^A126C-N152C^, towards *p*NPF, were 50.2 and 59.7 U/mg, respectively. SDS-PAGE assay displayed single protein bands of purified re-AuFaeA and re-AuFaeA^A126C-N152C^, with apparent molecular weights of approximately 36.0 kDa (Fig 4, lane 3 and 4).

### Formation of the Extra Disulfide Bridge

Introduction of the engineered disulfide bridge was verified by thiol titration of the purified re-AuFaeA and re-AuFaeA^A126C-N152C^ at concentrations of 2 mg/mL (0.056 mM) under the denaturing conditions. Based on the cysteine standard formula y = 0.8727x + 1.3972 (x: cysteine concentration, mM; y: OD_410_), the OD_410_ at the cysteine concentration of 0.056 mM was 1.446. The detected OD_410_ (1.452 and 1.437) of purified re-AuFaeA and re-AuFaeA^A126C-N152C^ were almost unanimous with the standard value (Table 2). Since re-AuFaeA containing one free cysteine at position 235 was used as the control, its absorption of 1.437 indicated that AuFaeA^A126C-N152C^ also had one free cysteine at the same position. In other words, with the exception of the native disulfide bridges, a new variant with an extra disulfide bridge C126-C152 was successfully created.

**Table 2:** Deduced number of free cysteines and disulfide bridges in re-AuFaeA and re-AuFaeA^A126C-N152C^.

Enzyme (2 mg/mL)	OD_410_ after DTNB treatment	Deduced number of free cysteines*	Deduced total number of disulfide bridges
re-AuFaeA	1.452	1	3
re-AuFaeA^A126C-N152C^	1.437	1	4

* Based on the cysteine standard formula y = 0.8727x + 1.3972 (x: cysteine concentration, mM; y: OD_410_), the OD_410_ at the cysteine concentration of 0.056 mM was 1.446.

### Thermal Properties of the re-AuFaeA and re-AuFaeA^A126C-N152C^

**Fig 5:**
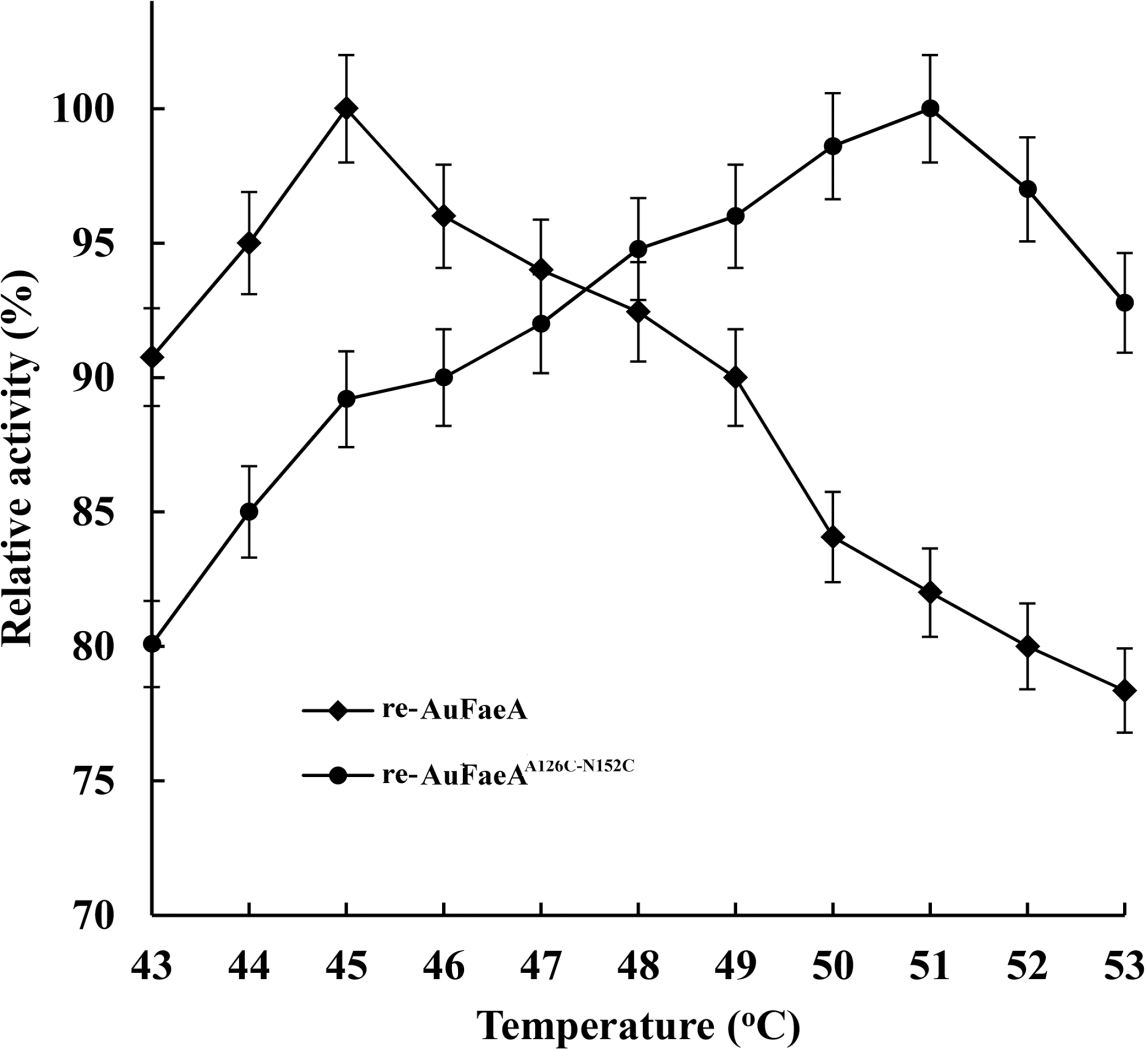
Temperature optima of the re-AuFaeA and re-AuFaeA^A126C-N152C^. The temperature optima were measured under the standard assay conditions, with variable temperatures ranging from 43 to 53°C.

**Fig 6:**
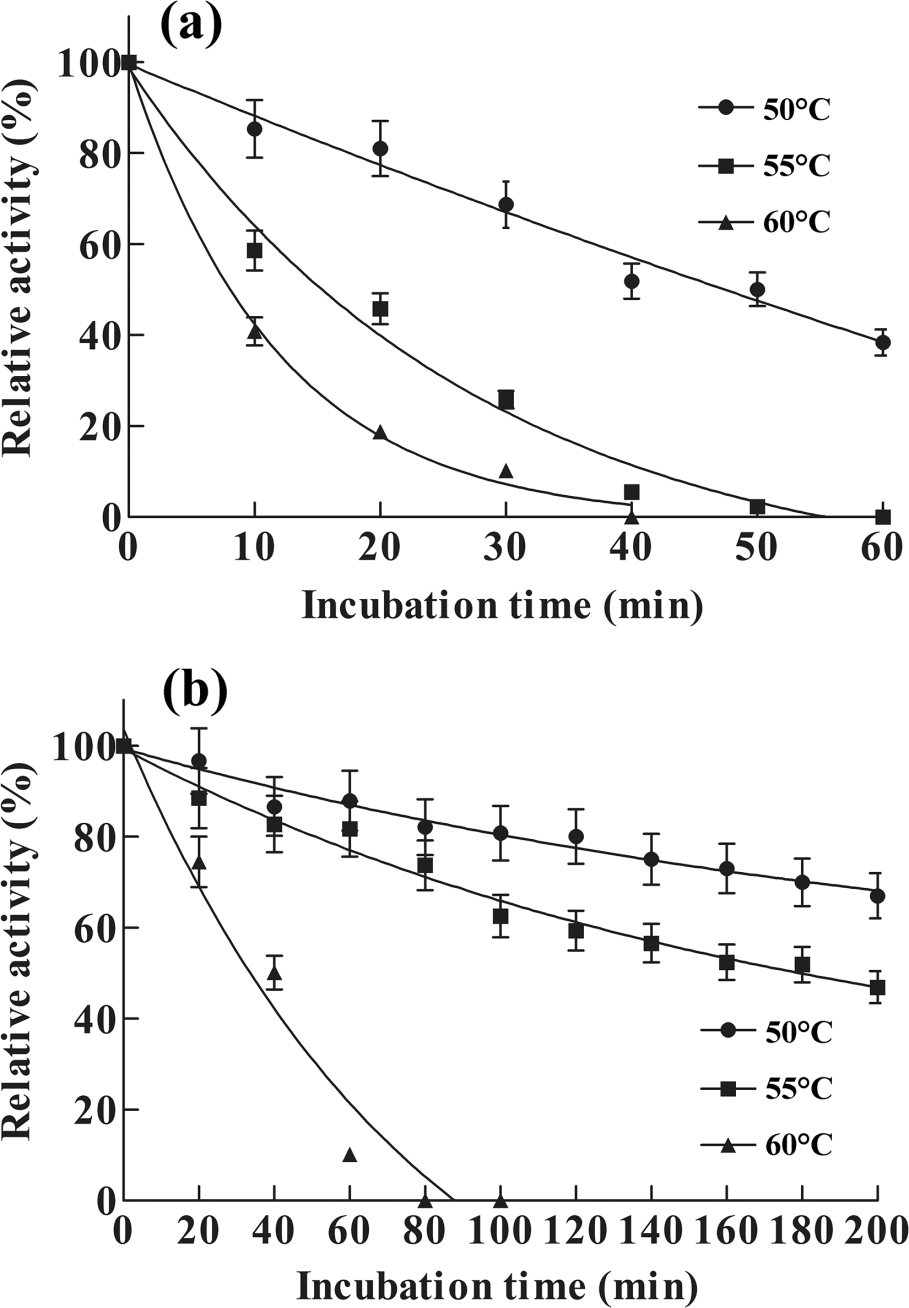
Thermostability of the re-AuFaeA and re-AuFaeA^A126C-N152C^. Thermostability of (a) re-AuFaeA and (b) re-AuFaeA^**A126C-N152C**^ were evaluated by incubating in the absence of substrate at temperatures ranging from 50 to 60°C for different time, respectively, and the residual enzyme activities were measured under the standard assay conditions.

The temperature optimum of re-AuFaeA^A126C-N152C^ was 51°C (Fig 5), which was 6°C higher than that of re-AuFaeA and even higher than those (43–47°C) of recombinant AnFaeA variants [17]. The thermal inactivation half-lives (*t*_1/2_) of re-AuFaeA at 50, 55 and 60°C were 50, 15 and 4 min (Fig 6A), respectively. It entirely lost its activity at 60°C for 40 min. The half-lives (*t*_1/2_) of re-AuFaeA^A126C-N152C^ at 55 and 60°C were 188 and 40 min, which were 12.5- and 10-folds longer than those of re-AuFaeA (Fig 6B), respectively. These results suggested that a properly engineered disulfide bridge is capable of improving the type A feruloyl esterase thermostability.

### Kinetic Parameters

The kinetic parameters of re-AuFaeA and re-AuFaeA^A126C-N152C^ were characterized with purified enzymes, using *p*NPF as the substrate (Table 3). The re-AuFaeA^A126C-N152C^ increased Michaelis constant (*K*_m_) by approximately 1.6-fold compared to that of the re-AuFaeA, indicating a possible decrease in substrate affinity. However, the re-AuFaeA^A126C-N152C^ also increased catalytic turnover frequency (*k*_cat_) by approximately 1.6-fold compared with that of re-AuFaeA. Consequently, the catalytic efficiency (*k*_cat_/*K*_m_) of re-AuFaeA^A126C-N152C^ was similar to that of re-AuFaeA. Since the kinetics of both enzymes were done at 40°C showing equal activity, the mutant enzyme was expected to show higher activity at higher temperature.

**Table 3:** Kinetic parameters based on Michaelis-Menten equation.

Enzyme	*K*_m_ (mM)	*V*_max_ (U mg^-1^)	*k*_cat_ (min^-1^)	*k*_cat_/*K*_m_ (mM^-1^ min^-1^)
re-AuFaeA	3.62±0.08	225±2.0	8093±72	2235±72
re-AuFaeA^A126C-N152C^	5.98±0.12	356±2.0	12805±72	2141±56

### Elimination of the Native Disulfide Bridge

It has been reported that, after removal of the disulfide bridge constraints on the Ribonuclease T1 flexibility, the enzyme’s folding and functional abilities were maintained, but its stability was decreased [42]. Similarly, it was deduced that the three native disulfide bridges, C29-C258, C91-94 and C227-C234 located in AuFaeA were most likely contributing to its thermostability. Using site-directed mutagenesis, three different mutants were generated each having one natural cysteine mutated to threonine.

**Fig 7:**
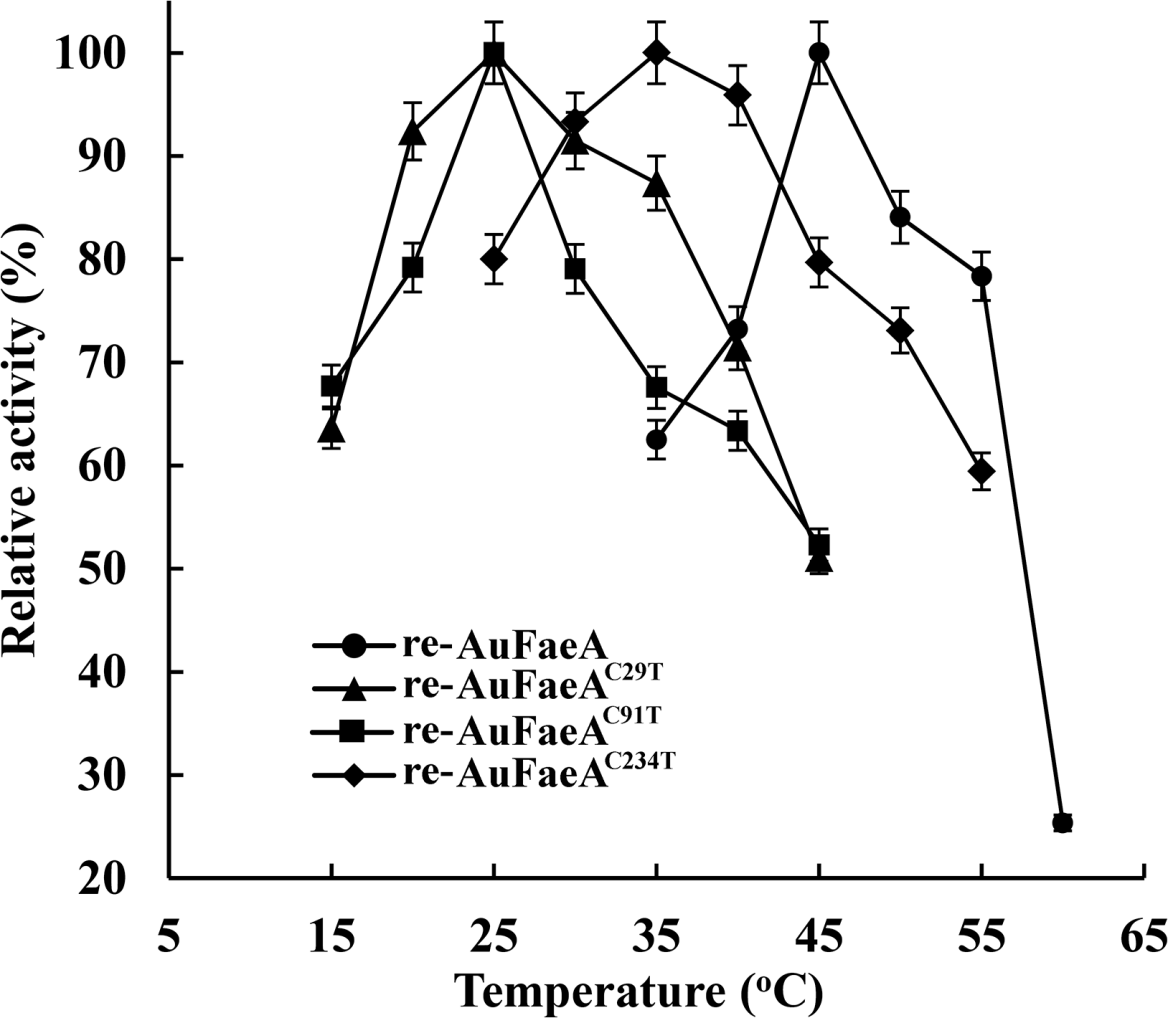
Temperature optima of the re-AuFaeA, re-AuFaeA^C29T^, re-AuFaeA^C91T^ and re-AuFaeA^C234T^. The temperature optima were measured under the standard assay conditions, with varying temperatures ranging from 15 to 60°C.

The activities of the expressed re-AuFaeA^C29T^, re-AuFaeA^C91T^ and re-AuFaeA^C234T^ in the cultured supernatants were up to 0.84, 0.42 and 0.45 U/mL, respectively, which were much lower than that (11.03 U/mL) of re-AuFaeA. The temperature optima of re-AuFaeA^C29T^, re-AuFaeA^C91T^ and re-AuFaeA^C234T^ were 25, 25 and 35°C, respectively (Fig 7), which were at least 10°C lower than that of re-AuFaeA. Their thermostabilities were also significantly decreased (below 25°C). These results demonstrated the importance of each native disulfide bridge to the thermostability of re-AuFaeA.

### Analysis of the 3D Structures of AuFaeA and Its Variants

The predicted 3D structures of AuFaeA and AuFaeA^A126C-N152C^ consist of a major nine-stranded mixed β-sheet, one minor two-stranded β-sheet arrangement and seven helixes. Three conserved residues in AuFaeA, viz. S133, D194 and H247, constitute a characteristic catalytic triad as that in AnFaeA [11], in which S133 acts as the nucleophile, H247 as the proton acceptor/donor and D194 as the residue stabilizing the histidine. Three native disulfide bridges located in AuFaeA were expected to stabilize its 3D structure from three directions (Fig 1A). Therefore, after the elimination of each native disulfide bridge, the structural conformation of AuFaeA was changed. The region was no longer rigid relatively, which resulted in the significantly decreased thermostability of AuFaeA. Inevitably, the introduced threonine could affect protein stability, because it introduces an additional methyl group, which could cause steric clashes that could further destabilize the enzymes. However, compared to the effect from the elimination of the native disulfide bridges on the stability of AuFaeA, the effect from the introduced methyl group could be weak. The results indicated that native disulfide bridges in type A FAEs from fungi could play an irreplaceable role in the protein stability.

**Fig 8:**
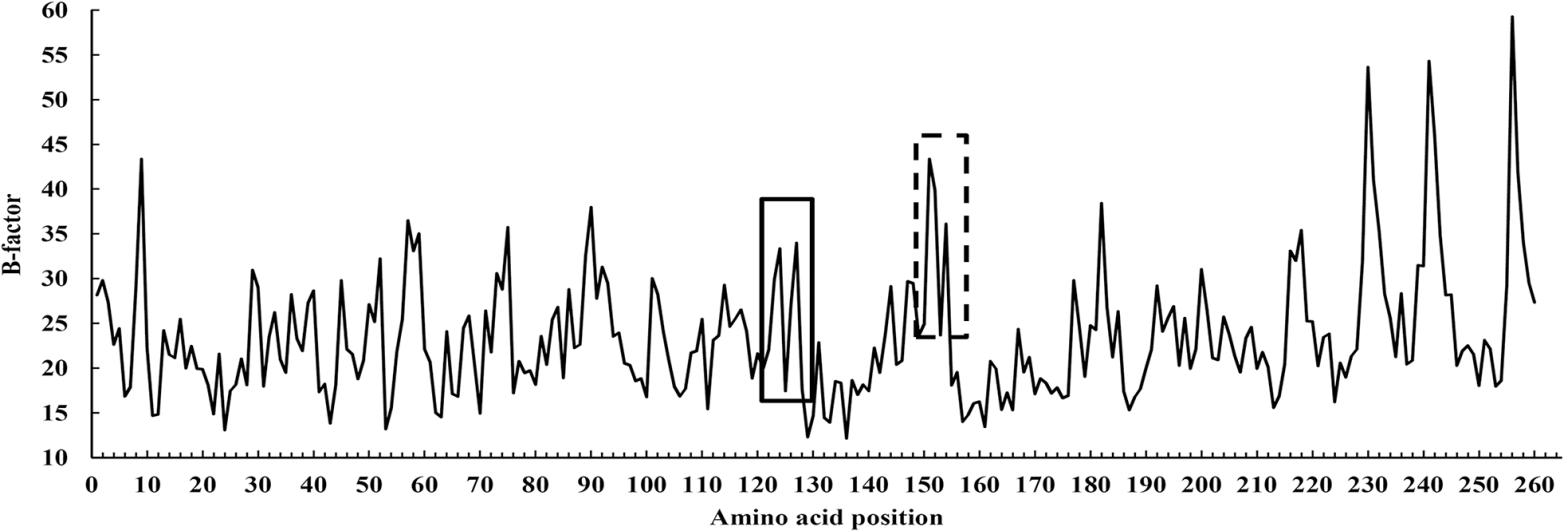
The B-factor values of amino acid residues of AnFaeA (PDB code: 1USW). The regions around amino acid residues 126 and 152 with high B-factor values are marked in solid and dashed boxes.

Compared the native disulfide bridges to the engineered one in the AuFaeA, the native bridges mainly stabilize regions with long loops, while the engineered C126-C152 binds the end of the β5 and β6 strands and could thus stabilize the edge of the large β-sheet (Fig 1B). This indicates that at high temperature the protein may unfold from the edge of the β-sheet but that this could be prevented by the disulfide bridge. Additionally, the B-factor values, namely the atomic displacement parameters, of AnFaeA (1USW) amino acid residues were analyzed by B-FITTER software (Fig 8). Compared the B-factors of engineered and native disulfide bridge positions in 1USW, the regions around amino acid sits 126 and 152 have high B-factor values, which indicates that these regions are more mobile. This fits into the strategy of stabilizing proteins by making mutations in regions with high B-factors [28]. It could be deduced that the engineered disulfide bridges in weak spots of the FAEs or other proteins, to some extent, could make them much more thermostable.

## Conclusions

Effects of disulfide bridges on the thermostability of AuFaeA were investigated by either introducing an extra disulfide bridge to, or by eliminating each native disulfide bridge from AuFaeA. Firstly, five pairs of amino acids (A24-R66, Y80-P200, F176-Y186, Y122-Y125 and A126-N152) that can form disulfide bridges in AuFaeA with the highest possibility were located by both MODIP and DbD. Then, the variant AuFaeA^A126C-N152C^ obtained by mutating A126 and N152 to C126 and C152 was predicted to be more thermostable than the wild-type AuFaeA by MD simulation. Finally, AuFaeA, AuFaeA^A126C-N152C^ and three variants (AuFaeA^C29T^, AuFaeA^C91T^ and AuFaeA^C234T^) each eliminating a native disulfide bridge were expressed in *P*. *pastoris* GS115. Experimental results with the disulfide bridge mutants confirmed that the disulfide bridges contribute significantly to the thermostability of AuFaeA. The computational prediction combined with site-directed mutagenesis to enhance the thermostability and to explore the thermostable mechanism of AuFaeA also can be applied in other proteins or enzymes.
